# Understanding complex biogeographic responses to climate change

**DOI:** 10.1038/srep12930

**Published:** 2015-08-06

**Authors:** Rui Seabra, David S. Wethey, António M. Santos, Fernando P. Lima

**Affiliations:** 1 CIBIO/InBIO, Centro de Investigação em Biodiversidade e Recursos Genéticos, Universidade do Porto, Campus Agrário de Vairão, 4485-661, Vairão, Portugal; 2 Departamento de Biologia, Faculdade de Ciências da Universidade do Porto, R. Campo Alegre, s/n, 4169-007 Porto, Portugal; 3 Department of Biological Sciences, University of South Carolina, Columbia, SC 29208

## Abstract

Predicting the extent and direction of species’ range shifts is a major priority for scientists and resource managers. Seminal studies have fostered the notion that biological systems responding to climate change-impacted variables (e.g., temperature, precipitation) should exhibit poleward range shifts but shifts contrary to that expectation have been frequently reported. Understanding whether those shifts are indeed contrary to climate change predictions involves understanding the most basic mechanisms determining the distribution of species. We assessed the patterns of ecologically relevant temperature metrics (e.g., daily range, min, max) along the European Atlantic coast. Temperature metrics have contrasting geographical patterns and latitude or the grand mean are poor predictors for many of them. Our data suggest that unless the appropriate metrics are analysed, the impact of climate change in even a single metric of a single stressor may lead to range shifts in directions that would otherwise be classified as “contrary to prediction”.

Changes in the distributional ranges of species are among the expected outcomes of climate change^1^. Several comprehensive studies report a broad prevalence of range shifts at poleward or upper range boundaries consistent with climate change predictions^2^,^3^,^4^,^5^,^6^,^7^. Still, shifts contrary to that expectation have been frequently reported^8^,^9^,^10^,^11^. Range shifts contrary to predictions may occur because organisms are responding to a different variable (related or unrelated to climate change), or because the predicted direction was wrongly established to begin with. In fact, by identifying appropriate controlling stressors, and refining how the predicted direction of change is established, recent analyses have shown that species may be tracking climate change even when distribution ranges are shifting in otherwise unexpected directions^12^,^13^,^14^. Making sense of shifts contrary to predictions is important as it may impact the confidence level of climate change attribution, and therefore influence public opinion and policy.

It is well recognized that the mean is a metric that oversimplifies much of the complexity of stressors^15^,^16^,^17^,^18^, and specific aspects of some stressors (e.g., minimum water temperature during winter) have been identified as playing key roles in determining biogeographic patterns^19^,^20^. However, since environmental data at the appropriate temporal and spatial scales are often lacking, the patterns of many aspects of stressors remain largely uncharacterized with appropriate detail. To fill this void, general perception seems to hold that whatever spatial gradient is detected in the mean must be reflected to a large extent by all other metrics, especially for cases where the mean neatly fits preconceived assumptions. There is, however, no statistical ground supporting that view, and we argue that this misunderstanding may lead to improper estimation of what exactly is the predicted direction of range shifts of a particular species as a response to changes in the patterns of a single stressor. This effect should be especially noticeable for systems following Liebig’s law of the minimum to some extent, which emphasizes the role of the scarcest resource (or, in this case, the least favourable bioclimatic variable) in determining habitat suitability at a given location and time.

Using temperature extremes as an example, the present study aims at characterising the contrasting patterns encapsulated within a single stressor, as well as showing that climate change-induced alterations of aspects of that stressor can potentially lead to otherwise non-intuitive range shifts.

## Results and Discussion

**Figure 1:**
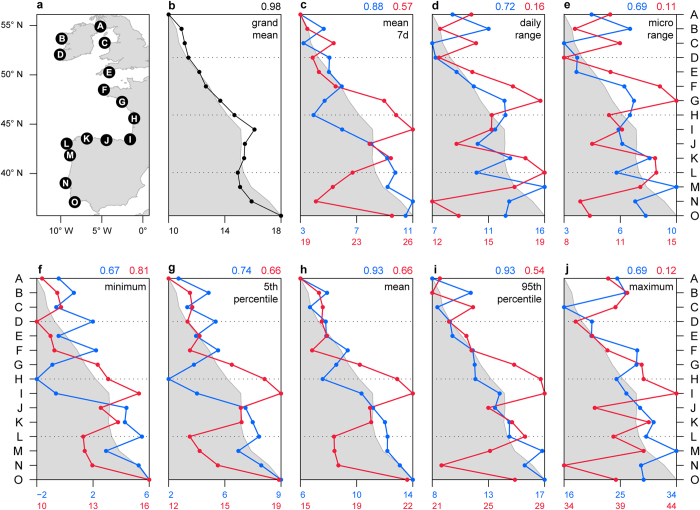
Patterns of temperature metrics across the European Atlantic intertidal ecosystem. (**a**) Locations surveyed. Geographic pattern of metrics: (**b**) grand mean, (**c**) 7 day mean, (**d**) daily range, (**e**) microhabitat range, (**f**) minimum, (**g**) 5^th^ percentile, (**h**) mean, (**i**) 95^th^ percentile, (**j**) maximum. Black line (**b**) is grand mean, calculated using all data from each shore. Red and blue lines (**c–j**) calculated using the warmest and coldest 30 days of each year (7 days for (**c**)), per shore. The shaded area is the pattern expected if each metric was perfectly correlated with latitude. Points in shaded area are “cooler than expected given latitude”, and points outside shaded area are “hotter than expected”. Correlation coefficients between each metric and latitude are depicted in the top right corner of each panel (blue for cold and red for warm periods). Map created in R^35^ using Global Self-consistent, Hierarchical, High-resolution Geography Database (GSHHG) coastline data.

We used a dataset comprised of 90 individual 4-year-long temperature time series (six microhabitats on 15 shores from 37 °N to 55 °N latitude, Fig. 1a) to evaluate to what extent the patterns of ecologically relevant metrics of a stressor are indeed captured by that stressor’s mean. Extreme temperature is a major stressor in most ecosystems, and especially in the rocky intertidal^16^, where animals and plants a few centimetres apart can be experiencing dramatic differences in body temperature^21^,^22^. The overall mean temperature, which is often used as a reference for estimating species distributions^17^, was shown to match latitude surprisingly well (R = 0.98, Fig. 1b), thus reinforcing the idea of a relatively smooth, continuous gradient from warmer to colder temperatures with increasing latitudes along the European Atlantic coastline. However, with the exception of ‘winter mean’ and ‘winter 95^th^ percentile’ (Fig. 1h,i, blue lines), all other metrics (‘summer mean’, ‘summer 95^th^ percentile’, and winter and summer ‘7 day mean’, ‘daily range’, ‘microhabitat range’, ‘minimum’, ‘5^th^ percentile’ and ‘maximum’) exhibited patterns deviating substantially from that of the grand mean. These differences highlight the key role played by climatic, geomorphologic and oceanographic factors at the local level, and more importantly, show that such factors can change skewness or kurtosis of the distribution of temperatures (or even causing multimodality) without affecting the mean. For example, seasonality, which is strongest within the Bay of Biscay — shores H and I — appears not to drive the grand mean for these shores too far away from the expected value given their latitude, but results in remarkably high summer temperatures (in fact the highest recorded in the study area; Fig. 1i,j, red lines) and equally remarkably low temperatures during winter (again, the lowest within the study area; Fig. 1f,g, blue lines). In another example, upwelling, which is typically stronger around shores F and L during summer^23^, can be seen driving ‘summer daily range’ and ‘summer microhabitat range’ (Fig. 1d,e, red lines), likely due to the co-occurrence of low water temperatures and high air temperatures. Again, this effect does not result in any important deviation of the grand mean from the latitudinal pattern for these shores, and would be largely missed if data at the appropriate spatial scale had not been collected. Additionally, the combination of regional factors and local context can result in surprising temperature distributions, such as seen at shore N. At this shore, all metrics were found to be lower or equal to the expected value based on latitude. However, the grand mean does not reflect the magnitude of this difference, especially considering that shore N is the coldest in the study area for some of the metrics calculated. The many patterns encapsulated within the distribution of values of a single stressor clearly indicates that the grand mean may largely misrepresent many other ecologically relevant aspects of that stressor. This is in accordance with previous studies^21^,^24^ and reinforces the notion that a-priori knowledge of the physiological requirements of a species and a detailed characterisation of the thermal extremes at the study area are fundamental to ascertain the real stress landscape imposed on organisms.

**Figure 2:**
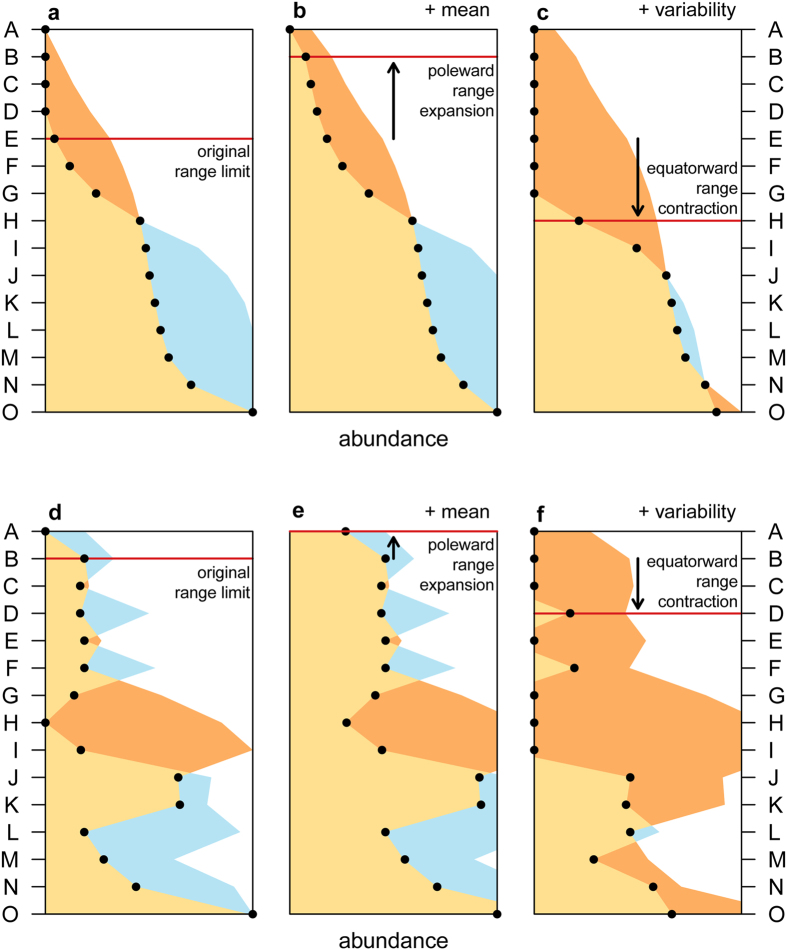
Climate change can generate complex biogeographic responses. Conceptual framework (**a–c**) and example built using real temperature data (**d–f**) illustrating the mechanism through which climate change may induce complex biogeographic responses. Black dots show the abundance of a hypothetical species in each location (A–O, see Fig. 1a), which results from the interplay of ‘winter minimum’ (blue areas) and ‘summer 5^th^ percentile’ (dark orange areas). Light orange results from the overlap between blue and orange areas and shows the outcome of the Liebig’s law of the minimum. (**a**,**d**) show the initial conditions, (**b**,**e**) result from the monotonic increase of both winter minimum and summer 5^th^ percentile (scenario of increased mean), and (**c**,**f**) from increase of one aspect of temperature and decrease of the other (scenario of increased variability but stable mean).

Furthermore, using a theoretical example we show that complex biogeographic responses to climate change can be interpreted by using the appropriate metrics (Fig. 2). We assume that the distribution of a theoretical species is determined by a group of relevant metrics (in this case the thermal extremes measured as ‘winter minimum’ and ‘summer 5^th^ percentile’) and follows Liebig’s law of the minimum (i.e., at each location density is dependent on the least favourable relevant metric). In the simplest form, the distribution pattern will be determined by the least favourable of a number of relevant metrics (Fig. 2a, light orange area). If climate change results in a favourable monotonic change of all metrics (Fig. 2b), the extent of suitable locations increases and a range expansion can be expected — the “general perception” poleward scenario. However, studies have highlighted that climate change not only can result in increased mean, minimum and maximum temperatures but also in increased variability — and that the exact signature of climate change varies regionally^18^,^25^,^26^,^27^. In this case, if at least one metric becomes less favourable due to the increased variability, the whole distribution can be adversely affected, and an equatorward range contraction may occur (Fig. 2c). Using the metrics computed in this study it is possible to further expand the example. If the distribution of a species was found to be dependent on the interplay between extremes like ‘winter minimum’ and ‘summer 5^th^ percentile’ the initial distribution pattern should include a gap at shore H, and a polar range limit at shore B (Fig. 2d). If both ‘winter minimum’ and ‘summer 5^th^ percentile’ become warmer, a poleward range expansion can be expected (Fig. 2e), but if ‘summer 5^th^ percentile’ becomes warmer while ‘winter minimum’ becomes colder, the harshness of winter conditions prevail over the favourable summers and a equatorward range contraction should occur (Fig. 2f). Interestingly, in a few locations suitability would actually increase because the limiting factor was ‘summer 5^th^ percentile’ and not ‘winter minimum’ (shores L and N, Fig. 2h), highlighting the consequences of different mechanisms limiting species’ densities across different locations^28^. The crucial point is that if field surveys were to reveal an equatorward range contraction for this species, this range shift would not be contrary to predictions, as general perception would suggest. Instead, it would be consistent with the predicted direction of change for this biological system’s response to climate change, thus representing positive evidence towards the establishment of a link to climate change.

In addition, it is conceivable that some organisms’ physiological requirements may include more complex interactions of aspects of a stressor than those depicted here^29^,^30^. For example, a mobile organism will be able to explore the various microhabitats available within a site for thermoregulation. If that organisms’ physiology is found to be negatively impacted by ‘summer maximum’, it is likely that the appropriate metric to study will instead be the difference between ‘summer maximum’ and ‘summer microhabitat range’, as very high temperatures can be avoided if cooler microhabitats are available. These complex interactions between aspects of a stressor can generate new stress landscape patterns that do not match that of the grand mean, ‘summer maximum’ or ‘summer microhabitat range’, further increasing the likelihood of erroneous expectations about the extent and direction of range shifts in face of climate change if the stress landscape is not properly characterized. The findings presented in this study reinforce the notion that using the appropriate metrics of a stressor and identifying the appropriate stressor^13^ can provide decisive insights towards the detection, interpretation and prediction of complex distribution patterns, spatial and temporal variations of mechanisms controlling species distributions and the direction of range shifts. In addition, the conceptual framework here outlined emphasizes the paramount importance of coupling the collection of environmental data at the appropriate scales with a detailed characterization of species’ physiological requirements (see Ashcroft *et al.*^31^ or Greenberg *et al.*^32^ for analogous approaches using modelled data). Although focused on the thermal regimes of the European Atlantic intertidal ecosystem, the concepts here outlined can be extended to other geographical regions, ecosystems, and stressors. Taken with necessary caution (see, for example, the cautionary advice by Austin^33^ regarding assumptions of linear response to temperature), these results suggest that some of the cases where species have been shown to be shifting in directions contrary to expectations, the predicted direction of change may have been wrongly established.

## Methods

### Microhabitat temperature

Intertidal microhabitat temperatures were recorded at 15 exposed to moderately exposed shores along the European Atlantic coast, spanning nearly 20° of latitude, from Southwest Scotland to South Portugal (Fig. 1a, A – South Cairn, B – Emlagh, C – Holyhead, D – Annascaul, E – Wembury, F – Landunvez, G – Batz-sur-Mer, H – Royan, I – Biarritz, J – Prellezo, K – La Caridad, L – Cabo Touriñan, M – Moledo, N – São Lourenço, O – Evaristo). Data were acquired using robolimpets (autonomous temperature sensing/logging devices mimicking the visual aspect and temperature trajectories of real limpets, see Lima and Wethey^34^ for details). Loggers were deployed following Seabra *et al.*^22^. Temperatures were sampled from 6 distinct combinations of height above the low water mark (low, mid and high shore) and exposure to sun (shaded and sun-exposed), thus covering most of the spectrum of microhabitats occupied by intertidal species. Data were collected continuously between the summers of 2010 and 2014, at a sampling rate of 60 minutes and a resolution of 0.5 °C. For each microhabitat, logged temperatures were averaged whenever data from multiple sensors were available. All data manipulation and analyses were done using R 3.1.2^35^.

### Data analysis

A total of 17 ecologically relevant temperature metrics were computed for each shore. Metrics were computed per year and then averaged over the four years of data available. Metrics computed include the mean using all available data for each shore (grand mean), the mean temperature during the hottest/coldest seven days of each year (‘mean 7d’), and the mean daily range of temperatures (‘daily range’), mean daily range of all microhabitats’ maximum temperatures (i.e., microhabitat range, or ‘micro range’), minimum, 5^th^ percentile, mean, 95^th^ percentile and maximum during the hottest/coldest 30 days of each year. For easier terminology, metrics computed during the hottest periods were prefixed “summer”, and metrics computed during the coldest periods were prefixed “winter” (e.g., ‘summer minimum’, ‘winter mean 7d’, etc.). The correlation coefficient between each metric and latitude was also calculated.

### Direction of range shifts

An example is presented to illustrate how range shifts driven by climate change can occur both towards the poles or the equator. We modelled the relative abundance (from 0 – absent, to 1 – highest abundance) of a theoretical species under two climate change scenarios. We modelled an equatorial species which is intolerant of cold stress and tolerant of heat stress. Abundance was determined as the lowest value of either ‘winter minimum’ or ‘summer 5^th^ percentile’ at each shore (following Liebig’s law of the minimum). To allow the comparison of both metrics (which are not equivalent in absolute terms) we normalised each metric to vary from 0 to 1, reflecting the range observed within the study region. Zero abundance occurred at shores where at least one of the metrics had a value of zero, meaning that either ‘winter minimum’ or ‘summer 5^th^ percentile’, or both, prevented the species from existing. Both metrics were used without any change for the initial conditions. The first climate change scenario considers climate change as a monotonic increase of both aspects of temperature, and the abundance pattern was computed using “hot” versions of both ‘winter minimum’ and ‘summer 5^th^ percentile’ (resulting in increased habitat suitability). The second scenario considers climate change as an increase of variability in which some metrics may actually become colder. In this case the abundance pattern was computed using the “cold” version of ‘winter minimum’ and the “hot” version ‘summer 5^th^ percentile’. The initial range limit was identified as the most poleward shore with abundance greater than zero. The location of the range limit was re-evaluated for both climate change scenarios to determine the direction of change (poleward or equatorward).

## Additional Information

**How to cite this article**: Seabra, R. *et al.* Understanding complex biogeographic responses to climate change. *Sci. Rep.* **5**, 12930; doi: 10.1038/srep12930 (2015).

## References

[b1] IPCC. Climate Change 2014: Impacts, Adaptation, and Vulnerability. Part A: Global and Sectoral Aspects. Contribution of Working Group II to the Fifth Assessment Report of the Intergovernmental Panel of Climate Change. (Cambridge University Press, 2014).

[b2] Parmesan C. & Yohe G. A globally coherent fingerprint of climate change impacts across natural systems. Nature 421, 37–42 (2003). 12511946 10.1038/nature01286

[b3] Walther G. E. *et al.* Ecological responses to recent climate change. Nature 416, 389–395 (2002). 11919621 10.1038/416389a

[b4] Fingerprints of global warming on wild animals and plants. 421, 57–60 (2003). 10.1038/nature01333 12511952

[b5] Hickling R., Roy D. B., Hill J. K., Fox R. & Thomas C. D. The distributions of a wide range of taxonomic groups are expanding polewards. Glob Change Biol 12, 450–455 (2006).

[b6] Lima F. P., Ribeiro P. A., Queiroz N., Hawkins S. J. & Santos A. M. Do distributional shifts of northern and southern species of algae match the warming pattern? Glob Change Biol 13, 2592–2604 (2007).

[b7] Poloczanska E. S. *et al.* Global imprint of climate change on marine life. Nature Clim Change 3, 919–925(2013).

[b8] Hilbish T. J. *et al.* Historical changes in the distributions of invasive and endemic marine invertebrates are contrary to global warming predictions: the effects of decadal climate oscillations. J Biogeogr 37, 423–431 (2010).

[b9] Goatley C. H. R. & Bellwood D. R. Moving towards the equator: reverse range shifts in two subtropical reef fish species, *Chromis nitida* (Pomacentridae) and *Pseudolabrus guentheri* (Labridae). Mar Biodivers Rec 7, e12 (2014).

[b10] Chen I. C., Hill J. K., Ohlemuller R., Roy D. B. & Thomas C. D. Rapid range shifts of species associated with high levels of climate warming. Science 333, 1024–1026 (2011). 21852500 10.1126/science.1206432

[b11] Moritz C. *et al.* Impact of a century of climate change on small-mammal communities in Yosemite National Park, USA. Science 322, 261–264 (2008). 18845755 10.1126/science.1163428

[b12] Burrows M. T. *et al.* The pace of shifting climate in marine and terrestrial ecosystems. Science 334, 652–655 (2011). 22053045 10.1126/science.1210288

[b13] VanDerWal J. *et al.* Focus on poleward shifts in species’ distribution underestimates the fingerprint of climate change. Nature Clim Change 3, 239–243 (2013).

[b14] Crimmins S. M., Dobrowski S. Z., Greenberg J. A., Abatzoglou J. T. & Mynsberge A. R. Changes in climatic water balance drive downhill shifts in plant species’ optimum elevations. Science 331, 324–327 (2011). 21252344 10.1126/science.1199040

[b15] Boer Den P. J. Spreading of risk and stabilization of animal numbers. Acta Biotheor 18, 165–194 (1968). 4984481 10.1007/BF01556726

[b16] Helmuth B., Mieszkowska N., Moore P. & Hawkins S. J. Living on the edge of two changing worlds: forecasting the responses of rocky intertidal ecosystems to climate change. Annu Rev Ecol Evol Syst 37, 373–404 (2006).

[b17] Helmuth B. *et al.* Beyond long-term averages: making biological sense of a rapidly changing world. Climate Change Responses 1, 6 (2014).

[b18] Vasseur D. A. *et al.* Increased temperature variation poses a greater risk to species than climate warming. Proceedings of the Royal Society B: Biological Sciences 281, 20132612 (2014). 24478296 10.1098/rspb.2013.2612 PMC3924069

[b19] Helmuth B. *et al.* Organismal climatology: analyzing environmental variability at scales relevant to physiological stress. J Exp Biol 213, 995–1003 (2010). 20190124 10.1242/jeb.038463

[b20] Wethey D. S. *et al.* Response of intertidal populations to climate: effects of extreme events versus long term change. J Exp Mar Biol Ecol 400, 132–144 (2011).

[b21] Helmuth B. *et al.* Mosaic patterns of thermal stress in the rocky intertidal zone: implications for climate change. Ecol Monogr 76, 461–479 (2006).

[b22] Seabra R., Wethey D. S., Santos A. M. & Lima F. P. Side matters: microhabitat influence on intertidal heat stress over a large geographical scale. J Exp Mar Biol Ecol 400, 200–208 (2011).

[b23] Lemos R. T. & Pires H. O. The upwelling regime off the west Portuguese coast, 1941-2000. Int J Climatol 24, 511–524 (2004).

[b24] Lathlean J. A., Ayre D. J. & Minchinton T. E. Estimating latitudinal variability in extreme heat stress on rocky intertidal shores. J Biogeogr 41, 1478–1491 (2014).

[b25] Easterling D. R. *et al.* Climate extremes: Observations, modeling, and impacts. Science 289, 2068–2074 (2000). 11000103 10.1126/science.289.5487.2068

[b26] Lima F. P. & Wethey D. S. Three decades of high-resolution coastal sea surface temperatures reveal more than warming. Nat Commun 3 (2012). 10.1038/ncomms1713 22426225

[b27] IPCC. Climate Change 2013: The Physical Science Basis. Contribution of Working Group I to the Fifth Assessment Report of the Intergovernmental Panel on Climate Change. (Cambridge University Press, 2013).

[b28] Woodin S. A., Hilbish T. J., Helmuth B., Jones S. J. & Wethey D. S. Climate change, species distribution models, and physiological performance metrics: predicting when biogeographic models are likely to fail. Ecol Evol 3, 3334–3346 (2013). 24223272 10.1002/ece3.680 PMC3797481

[b29] Magozzi S. & Calosi P. Integrating metabolic performance, thermal tolerance, and plasticity enables for more accurate predictions on species vulnerability to acute and chronic effects of global warming. Glob Change Biol 21, 181–194 (2015). 10.1111/gcb.12695 25155644

[b30] Fu Y. H. *et al.* Increased heat requirement for leaf flushing in temperate woody species over 1980‐2012: effects of chilling, precipitation and insolation. Glob Change Biol 21, 2687–2697 (2015). 10.1111/gcb.12863 25580596

[b31] Ashcroft M. B., Cavanagh M., Eldridge M. D. B. & Gollan J. R. Testing the ability of topoclimatic grids of extreme temperatures to explain the distribution of the endangered brush‐tailed rock‐wallaby (*Petrogale penicillata*). J Biogeogr 41, 1402–1413 (2014).

[b32] Greenberg J. A., Santos M. J., Dobrowski S. Z., Vanderbilt V. C. & Ustin S. L. Quantifying Environmental Limiting Factors on Tree Cover Using Geospatial Data. PLOS ONE 10, e0114648 (2015). 25692604 10.1371/journal.pone.0114648 PMC4333833

[b33] Austin M. P. Spatial prediction of species distribution: an interface between ecological theory and statistical modelling. Ecol Model 157, 101–118 (2002).

[b34] Lima F. P. & Wethey D. S. Robolimpets: measuring intertidal body temperatures using biomimetic loggers. Limnol Oceanogr-Meth 7, 347–353 (2009).

[b35] R: A Language and Environment for Statistical Computing, R Core Team, R Foundation for Statistical Computing, Vienna, Austria, (2015) http://www.R-project.org.

